# Efficacy of virtual reality to reduce chronic low back pain: Proof-of-concept of a non-pharmacological approach on pain, quality of life, neuropsychological and functional outcome

**DOI:** 10.1371/journal.pone.0216858

**Published:** 2019-05-23

**Authors:** Federica Alemanno, Elise Houdayer, Daniele Emedoli, Matteo Locatelli, Pietro Mortini, Carlo Mandelli, Alberto Raggi, Sandro Iannaccone

**Affiliations:** 1 Department of Rehabilitation and Functional Recovery, IRCCS San Raffaele Hospital and Vita-Salute San Raffaele University, Milan, Italy; 2 Department of Neurosurgery and Gamma Knife Radiosurgery, IRCCS San Raffaele Hospital and Vita-Salute University, Milan, Italy; 3 Unit of Neurology, G.B. Morgagni – L. Pierantoni Hospital, Forlì, Italy; Anglia Ruskin University, UNITED KINGDOM

## Abstract

**Objectives:**

Chronic pain, such as low-back pain, can be a highly disabling condition degrading people’s quality of life (QoL). Not every patient responds to pharmacological therapies, thus alternative treatments have to be developed. The chronicity of pain can lead to a somatic dysperception, meaning a mismatch between patients’ own body perception and its actual physical state. Since clinical evaluation of pain relies on patients’ subjective reports, a body image disruption can be associated with an incorrect pain rating inducing incorrect treatment and a possible risk of drug abuse. Our aim was to reduce chronic low-back pain through a multimodal neurorehabilitative strategy using innovative technologies to help patients regain a correct body image.

**Methods:**

Twenty patients with chronic low-back pain were included. Before and after treatment, patients underwent: a neurological exam; a neuro-psychological evaluation testing cognitive functions (memory, attention, executive functions) and personality traits, QoL and mood; pain ratings; sensorimotor functional abilities’ testing. Patients underwent a 6 week-neurorehabilitative treatment (total 12 sessions) using virtual reality (VRRS system, Khymeia, Italy). Treatment consisted on teaching patients to execute correct movements with the painful body parts to regain a correct body image, based on the augmented multisensory feedback (auditory, visual) provided by the VRRS.

**Results:**

Our data showed significant reductions in all pain rating scale scores (p<0.05); significant improvements of QoL in the domains of physical functioning, physical role functioning, bodily pain, vitality, and social role functioning; improvements in cognitive functions (p<0.05); improvements in functional scales (p<0.05) and mood (p = 0.04).

**Conclusion:**

This non-pharmacological approach was able to act on the multi-dimensional aspects of pain and improved patients’ QoL, pain intensity, mood and patient’s functional abilities.

## Introduction

Chronic pain is defined as an unpleasant sensory and emotional experience associated with actual or potential tissue damage, or described in terms of such damage that has been lasting for at least twelve weeks [1]. Chronic pain, such as low back pain, is a highly disabling condition severely degrading people’s quality of life [2,3]. Epidemiological studies have found that 10.1% to 55.2% of people in various countries have chronic pain [4], which is now acknowledged as a condition in its own right [1]. Many patients do not respond to pharmacological treatment, inducing the clinical condition of chronic pain. Besides the unpleasant feeling, chronic pain may also affect cognitive and emotional functioning. Indeed, many studies demonstrated depressive symptoms and deficits in attention, verbal memory and executive functions within this syndrome [5]. It should also be said that altered personality profiles such as hysteria, hypochondria, depression or anxiety have been often reported following clinical measurements of personality traits in people with this condition [6–9]. All these notions lead to the conclusion that chronic pain, which refers to a multimodal experience, may contribute to disability, anxiety, depression, sleep disturbances, poor quality of life (QoL), and elevated healthcare costs [10–12].

It has also been demonstrated that chronic pain can lead to a somatic disperception, meaning a substantial mismatch between the sensation of the affected body part and its actual physical state [13–15]. Distortion of body image is widespread in painful disorders. It has been especially demonstrated in phantom limb pain, chronic low-back pain and Complex Regional Pain Syndrome (CRPS) [14–17]. In these studies, patients were found to have a distorted body image or, in some cases, a disruption of their self-perception. Since the clinical rating of pain relies on patients’ subjective reports, a distorted body image could lead to an incorrect pain rating, which could in turn lead to an incorrect pain treatment and to the risk of drug overuse.

Distortion of body image has been demonstrated in a majority of patients presenting with CRPS and low-back pain. Förderreuther and colleagues [15] studied a population of 114 patients with CRPS and reported that 54.4% of them felt their hand as “foreign” or “strange” and 48% of patients had impairments of identification of fingers of the affected hand. In chronic low-back pain, five out of six patients studied by Moseley et al. [16] could not delineate the full extent of their trunk, mentioning: “I can’t find it”. These reports are essentially built on patients’ own feeling about their painful body part. The definition of more objective outputs would be useful to better assess body image distortion.

Sensorimotor cortical reorganization has been demonstrated in chronic pain due to deafferentation [18], but also in chronic low-back pain [13] and CRPS [19]. Flor and colleagues showed an enhanced reactivity and a somatotopic reorganization of the somatosensory cortex in patients with chronic low back pain, hypothesizing an important role in the persistence of the painful experience [13]. Indeed, this cortical reorganization reversed with clinical improvement of painful sensations [20].

Several non-pharmacological approaches can be administered to reduce chronic low-back pain, such as massage, exercise, spinal manipulation, cognitive-behavioral therapy, acupuncture, yoga or functional restoration. These cognitive-behavioral, sensory or motor strategies aim particularly at reducing pain through cortical plasticity due to sensorimotor or cognitive stimulation. Most of these approaches aim to teach patients the best techniques to lead them to a self-management of their pain experience, through a manipulation of their thoughts, their feelings and their behavior. Such techniques have been demonstrated as moderately effective to reduce chronic low-back pain [21].

Virtual reality (VR) is a technological rehabilitation tool that allows the user to experience the interaction with a computer-generated environment [22]. It may provide some advantages over conventional care: it allows the simulation of realistic environments and real-life exercises; the activities can be personalized to meet the specific needs of the patient; patients feel more motivated by this kind of virtual environment [22–24]. In the rehabilitative context, motivation is an important factor influencing the performance outcome [25]. VR constitutes an enriched environment with augmented multiple sensory feedbacks (auditory, visual, tactile). Whit the usage of an enriched environment together with a moving avatar, VR rehabilitation engages several cortical and subcortical neuronal circuits that potentiate patient’s learning and recovery [26–28].

Thus, VR may be considered a good candidate to help patients in improving their own movements and body position perception [29] in order to regain a correct central nervous system body image. VR enriched environment has already shown some efficiency in reducing chronic pain either during the VR exercises [30] or after 3 to 10 days of rehabilitation [31]. These preliminary results provide evidence for safety and possible efficacy of this treatment. However, so far, no studies have investigated the effects of VR in longer periods.

In this proof-of-concept study, we treated chronic low back pain with a non-immersive virtual reality training starting from the working hypothesis that this treatment might contribute to restore a correct body image, improve QoL (primary outcome measure), reduce pain sensations, act positively on mood, and recover sensorimotor abilities.

## Materials and methods

### Participants

The study was conducted in the Neurorehabilitation and Functional Recovery Department of the San Raffaele Hospital (Milan, Italy). In this single-armed study, 20 patients (11 female, mean age 47.5 ± 15.3 y.o., age range [19–72]) presenting with chronic low back pain were included (mean pain duration 35.0 ± 42.4 months, range [3–144], see Table 1 for patients’ description). Inclusion criteria were: (1) age between 18 and 75 years, and (2) history of chronic pain ≥ 12 weeks. Exclusion criteria were: (1) systemic metabolic disorder, (2) neurological or muscular degenerative disorder, (3) systemic infection, (4) cardiopulmonary or pulmonary disorder with contraindication to physical exercise, (5) recent spinal surgery (<12 months), (6) spinal pathologies such as stenosis or spondylolisthesis or fracture, (7) acute radiculopathy or compromised nerve root, (8) pregnancy.

**Table 1:** Description of the sample.

Patients	Age	Gender	Pain duration
LBP-1	57	F	48
LBP-2	53	M	24
LBP-3	71	M	19
LBP-4	50	F	8
LBP-5	58	M	120
LBP-6	42	F	3
LBP-7	19	M	16
LBP-8	64	M	144
LBP-9	45	F	48
LBP-10	45	F	4
LBP-11	48	M	120
LBP-12	42	M	5
LBP-13	26	F	5
LBP-14	55	F	4
LBP-15	69	F	17
LBP-16	28	F	24
LBP-17	72	F	24
LBP-18	44	F	12
LBP-19	30	M	24
LBP-20	32	M	30

LBP: low-back pain. F: female, M: male. Age is expressed in years, and pain duration is expressed in months.

Most of the patients were referred to the Neurorehabilitation and Functional Recovery Department by the Neurosurgery and Gamma Knife Radiosurgery Department of the San Raffaele Hospital. All the patients had made a neurological visit and none of them had any indication for surgery. Patients had negative electromyographic evaluations for acute pathology. The study was approved by the local Ethics Committee of the San Raffaele Hospital (Milan, Italy) and all participants signed an informed consent according to the Declaration of Helsinki before entering the survey.

### Evaluations

Before and after treatment, subjects underwent a comprehensive neuropsychological assessment and a physical therapy examination.

In the neuropsychological assessment, the following tests for different cognitive domains and questionnaires for daily living activities were administered: (1) Activities of Daily Living (ADL) [32], (2) Instrumental Activities of Daily Living (IADL) [33], (3) Mini Mental State Examination (MMSE) [34], (4) Attentive and Raven Matrices [35], (5) Token test [36], (6) Semantic fluency [37], (7) Phonemic fluency [37], (8) naming [38], (9) word picture matching test [39], (10) Digit span test [40], (11) Digit Span Backward [41], (12) Corsi block-tapping test [42], (13) Rey Complex Figure Test [43], (14) Trail making test [44], (15) Stroop test [45], (16) Wisconsin Card Sorting test [46], (17) personality (Minnesota multiphasic personality inventory test 2 (MMPI-2, only in pre)) [47], (18) depression (Beck Depression Inventory-II) [48], (19) quality of life (SF36—Short Form Health Survey) [49]. This is a measure of health status contemplating eight sections: “vitality”, “physical functioning”, “bodily pain”, “general health perceptions”, “physical role functioning”, “emotional role functioning”, “social role functioning”, “mental health”[49,50].

In the physical therapy exam, the functional and pain assessment included: (1) an 11-point numeric rating scale (NRS) [51], (2) the McGill Pain Questionnaire (MPQ) [52], (3) the Brief Pain Inventoy (short form) (BPI) [53], and (5) the Roland and Morris Disability Questionnaire (RMDQ) [54]. Kinematic data were also measured using the Polhemus G4 tracking system and consisted in measuring the maximal and the average trunk’s range of motion during ten consecutive rotations, flexions, extensions and lateral flexions [55,56]. Maximal and Average Rotation reflect the range of motion during ten trunk’s rotations, expressed in degree. We also defined a Repetition Index, which is an index of proprioception ranging from 0 to 1, where 1 corresponds to the maximal accuracy in performing the same range of motion during ten trunk rotations. Participants wore 2 sensors: sensor 1 was positioned on the manubrium of the sternum and sensor 2 was positioned on the anterior superior iliac spine. The Euler Angles were measured by the differential orientation of the two sensors (relative movement of sensor 1 compared to the movement of sensor 2).

The Repetition Index was calculated as follows:

- Right rotation: IR+ = avg(Mn+)/max(Mn+)
- Left rotation: IR- = avg(Mn-)/min(Mn-),

Mn+ is defined as the Maximum Value of the all Positive Values of -n repetitions of the movement (right rotation) and Mn- is defined as the Minimum Value of all Negative Values of -n repetitions of the movement (left rotation).

Then, the total Repetition Index (IR) consisted on the average of IR+ and IR-.

After treatment, patients were also asked to report their Global Impression of Change (GIC) on a 7-point categorical scale [57].

### Treatments

Patients underwent 12 rehabilitative sessions of 1 hour each, over a period of 4 to 6 weeks. Treatments consisted in virtual reality-based sensorimotor rehabilitation provided by the Virtual Reality Rehabilitation System (VRRS) of the Khymeia group (Noventa Padovana, Italy). The technological equipment included a computer workstation connected to a 6 degrees of freedom (DOF) motion-tracking system (Polhemus G4, Vermont, US), a high-resolution LCD displaying the virtual scenarios on a large screen and a software processing the motion data. These data are issued by the receiver of the end-effector placed on the sternum or the hips.

**Fig 1:**
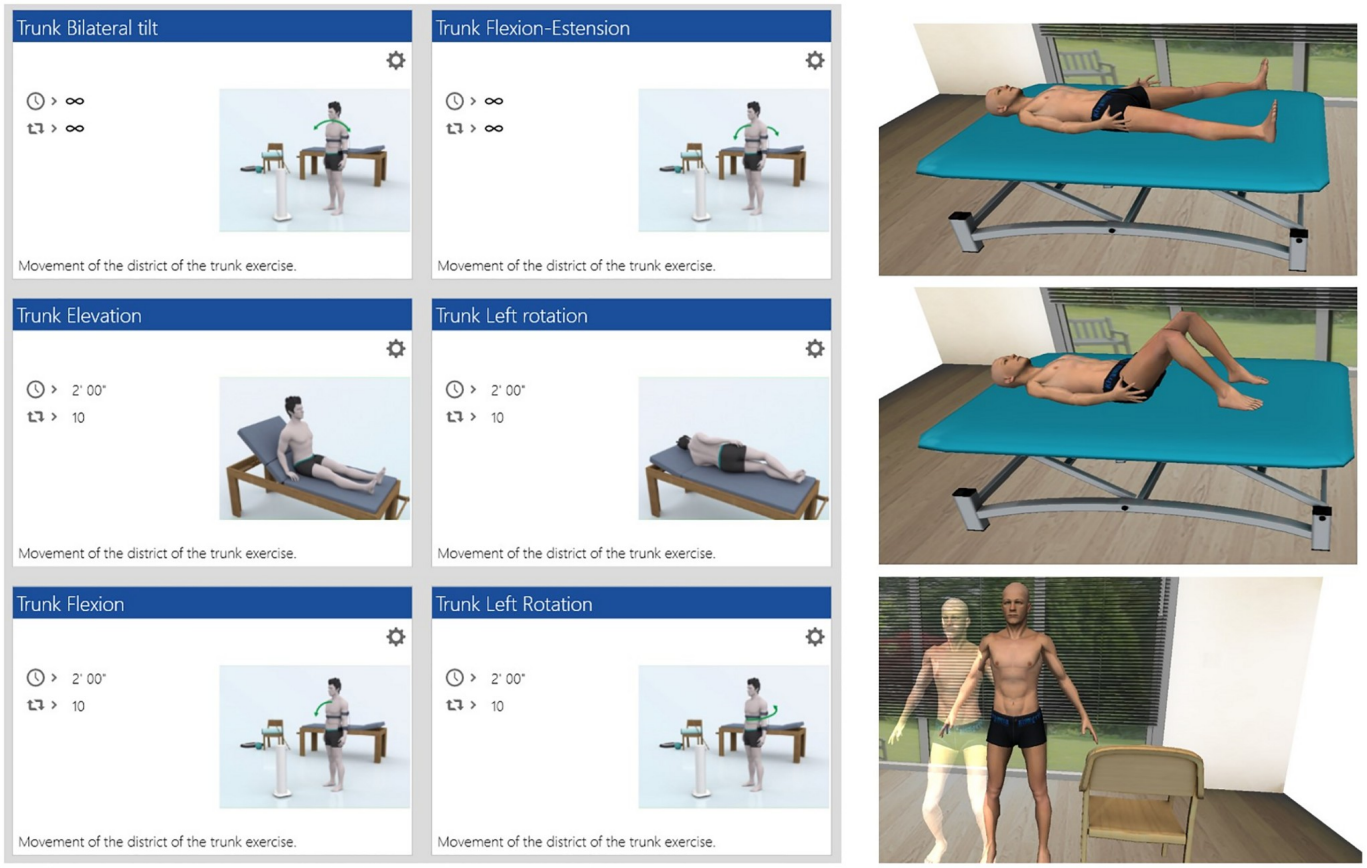
VR-based rehabilitation. Figure displays example zs of exercises performed by the participants using the virtual reality system. Subjects wore sensors on hips and/or sternum and were asked to perform movements in front of the computer where an avatar and virtual objects reproduced online the virtual movements.

The VRRS allows the participant to perform the requested motor tasks, while the movement of the system’s end-effector is simultaneously represented in a virtual scenario. In this scenario, an avatar reproduces online the performance of the patient who also gets an immediate visual and acoustic feedback on his/her performance. Indeed, for each exercise, the patient has to reach a certain result. The performance of the patient is immediately codified in terms of score, color code and acoustic feedback so that the patient always has a knowledge of performance and result. The aim of the exercises was to regain a correct body image by improving the control of single movements of the trunk. Patients underwent a series of exercises consisting mainly in trunk rotation, flexion and extension realized in various positions (standing, sitting, and kneeling) as displayed in Fig 1.

### Statistical analyses

Power size calculation was done on primary outcome: QoL, measured with the SF36—Short Form Health Survey. Considering a SF36 post-treatment change of 7.7 points and a standard deviation of 10.7[58], a 5% type I error (α = 0.05), 80% power (β = 0.2) and 15% of dropout rate, a sample size of 20 participants has been established (calculation was made with PS Power and Sample Size Calculation software). Pre and post values of all the pain ratings, neuropsychological and functional assessments were compared using either Student’s t-test for paired values or Wilcoxon test, depending on the normality of data distribution, as evaluated by the Shapiro-Wilk test. Correlations between improvements in pain and/or neuropsychological and functional scores (measured as differences between pre and post values) were tested using Pearson and Spearman correlations, depending on the data distribution.

Data were considered significant when p<0.05. The commercially available software IBM SPSS Statistics v.23 (IBM Corp.) was used for all statistical tests.

## Results

Every patient completed the sessions of virtual reality rehabilitation without side effects and with a good compliance (no dropouts during treatment). Seven out of the twenty patients refused to take part to some of the post-treatment neuropsychological evaluation. Thus, analyses of neuropsychological data are presented with n = 13 (except for QoL, BDI and MMPI, for which n = 20).

### Global improvement and pain ratings

Eighteen out of the twenty patients reported an improvement on the NRS pain scale (Table 2). Indeed, Wilcoxon test showed a significant improvement in NRS pain rating after treatment (p<0.001) overstepping also the Minimal Clinically Important Difference (MCID) set at 2.4 points in chronic low-back pain [59]. This improvement in NRS pain score correlated with the improvement reported by the patients on the GIC scale (Spearman R = -0.638, p = 0.002). Indeed, 16 patients reported improvement on the GIC scale (from slight to extreme improvements) and 3 patients reported an impression of no change. Significant improvements at the McGill Pain Questionnaire were also observed for the total pain score (Pain Rating Index-Total, PRI-TOT, p = 0.001) and the number of words used to described the pain (Number of Words Chosen, NWC, p = 0.001). The Brief Pain Inventory also reported significant improvements at the mean interference score (p<0.001), and at the worst and average pain scores (respectively, p = 0.002 and p<0.001), while no difference was observed for the least pain score (p = 0.077).

**Table 2:** Pain evaluations.

Tests		Pre	Post	P value
NRS pain		7.5 (5.0 to 8.38)	3.0 (1.63 to 6.5)	< 0.001 *
McGill Pain Questionnaire	PRI-TOT	31.85 ± 12.17	23.35 ± 16.70	0.001 *
	NWC	12.6 ± 4.56	9.05 ± 6.07	0.001 *
Brief Pain Inventory	Interference score	59.36 ± 20.45	34.70 ± 29.94	< 0.001 *
	Worst pain	66.02 ± 19.12	46.58 ± 31.0	0.002 *
	Average pain	55 (35 to 70)	35 (15 to 50)	< 0.001 *
	Least pain	30 (10 to 55)	5 (0 to 40)	0.077

Data are expressed in mean ± standard deviations or medians and (in parenthesis) first and third quartiles, depending on data normality. NRS: 11-point numeric rating scale; McGill Pain Questionnaire: PRI-TOT: Pain Rating Index-Total; NWC: Number of Words Chosen.

*: significant differences between pre and post values.

### QoL, mood, neuropsychological evaluation and psychological profile

Results are displayed in Table 3. The statistical analyses of the SF-36 revealed significant improvements in 5 out of the 8 the subscale scores. Indeed, significant improvements were observed in: (1) “physical functioning” (p = 0.018), “physical role functioning” (p = 0.04), (3) “bodily pain” (p = 0.029), (4) “vitality” (p = 0.015), and (5) “social role functioning” (p = 0.028). There was also a trend for an improvement in “emotional role functioning” (p = 0.062). There were significant correlations between the improvements in quality of life and improvements in pain scores. In particular, improvements in “physical functioning” correlated significantly with improvements in: (1) NRS scores (R = -0.521, p = 0.047), (2) McGill pain score PRI-TOT (R = -0.550, p = 0.034), and (3) BPI average pain (R = -0.673, p = 0.008) and worst pain (R = -0.563, p = 0.036). Improvements in “physical role functioning” correlated with improvements in: (1) McGill number of words chosen (NWC, R = -0.545, p = 0.036), and (2) BPI worst pain (R = -0.544, p = 0.044). Improvements in “vitality” correlated with BPI average pain improvements (R = -0.627, p = 0.016). Improvements in “social role functioning” correlated with: GIC scores (R = 0.599, p = 0.018), (2) improvements in NRS scores (R = -0.545, p = 0.036), (3) McGill PRI-TOT (R = -0.575, p = 0.025), and (4) BPI interference score (R = -0.650, p = 0.012).

**Table 3:** Evaluation of mood and quality of life.

Tests		Pre	Post	P value
BDI		13.5 (9.0 to 19.0)	3.5 (2.25 to 17.75)	0.037 *
SF-36	PF	49.67 ± 20.83	63.67 ± 20.04	0.018 *
	PR	0.0 (0.0 to 25.0)	0.0 (0.0 to 75.0)	0.040 *
	BP	28.53 ± 18.16	42.27 ± 22.38	0.029 *
	GH	50.67 ± 21.44	50.50 ± 25.76	0.966
	VT	40.67 ± 15.45	51.0 ± 18.92	0.015 *
	SR	44.97 ± 24.43	57.33 ± 31.27	0.044 *
	ER	0.0 (0.0 to 33.33)	33.30 (0.0 to 100)	0.062
	MH	57.87 ± 14.57	63.73 ± 22.80	0.183

Data are expressed in mean ± standard deviations or medians and (in parenthesis) first and third quartiles, depending on data normality. BDI: Beck Depression Inventory. SF-36: Short Form Health Survey. Results are exposed according to the 8 subscales: PF: Physical Functioning; PR: Physical Role Functioning; BP: Bodily Pain; GH: General Health; VT: Vitality; SR: Social Role functioning; ER: Emotional Role functioning; MH: Mental Health.

* shows significant differences between pre and post values.

Lastly, a significant post-treatment improvement of the Beck Depression Inventory-II scores was found (p = 0.04).

Neuropsychological evaluations did not show clinically significant abnormalities in patients’ cognitive functions. The statistical analyses revealed some significant improvements after treatment at the following tests (see Table 4): naming (p = 0.038), digit span (p = 0.013), Rey list immediate and late recall (respectively p = 0.024 and p = 0.008) and Stroop test with improvements in time (p = 0.004) and number of errors (p = 0.046). Moreover, there was a significant correlation between the improvement at the digit span scores and improvements in pain sensations (McGill, NWC, R = 0.591, p = 0.033).

**Table 4:** Neuropsychological evaluations.

Tests		Pre	Post	P value
ADL		6.00 (6.0 to 6.0)	6.00 (6.0 to 6.0)	0.317
IADL		8.0 (8.0 to 8.0)	8.0 (8.0 to 8.0)	0.317
MMSE		29.00 (28.5 to 30.0)	30.0 (28.5 to 30.0)	0.864
Token test		34.5 (32.5 to 35)	34.0 (32.0 to 35.5)	0.918
Semantic fluency		48.0 ± 11.11	49.25 ± 5.38	0.712
Phonemic fluency		33.75 ± 9.39	28.75 ± 6.08	0.138
Naming		47.0 (46.0 to 48.0) *	48.0 (47.0 to 48.0)*	0.038 *
Word picture matching test		46.75 ± 0.96	47.75 ± 0.5	1.00
Digit span test		6.0 (5.0 to 6.0) *	6.0 (5.5 to 7.0) *	0.013 *
Digit span backward		4.0 (3.0 to 5.0)	4 (4.0 to 5.0)	0.153
Corsi block-tapping test		5.0 (4.5 to 6.0)	6.0 (5.0 to 6.5)	0.190
Rey List Test	Immediate	42.75 ± 4.79 *	54.75 ± 9.71 *	0.024 *
	Deferred	10.0 ± 0.82	10.25 ± 1.71	0.508
	Recognition	14.5 (14.0 to 15.0)	15.0 (13.5 to 15.0)	0.860
Rey Figure	Late Recall	11.0 (7.0 to 14.5) *	14.5 (10.5 to 18.0) *	0.008 *
Raven’s Progressive Matrices		29.75 ± 3.10	30.50 ± 5.20	0.399
Attentional Matrices		53.50 ± 5.32	54.25 ± 3.40	0.301
Trail making test	A	37.75 ± 6.65	42.50 ± 6.14	0.605
	B	106.0 ± 35.19	95.25 ± 30.55	0.220
	B-A	58.0 (48.5 to 115.0)	55.0 (38.5 to 74.5)	0.239
Stroop test	Time	21.88 ± 6.73 *	16.25 ± 4.63 *	0.004 *
	Errors	0.0 (0.0 to 1.0) *	0,0 (0.0 to 0.0) *	0.046 *
Wisconsin Card Sorting test	Total	70.25 ± 61.16	63.0 ± 47.90	0.346
	Preservative errors	18.75 ± 17.15	14.25 ± 9.54	0.259
	Non preservative errors	19.50 ± 17.02	19.0 ± 18.53	0.702
	Failures	2.25 ± 2.63	2.75 ± 1.50	0.309
Rey figure copy		34.25 ± 0.96	33.75 ± 0.96	0.307

Table displays pre and post values expressed in mean ± standard deviations or in medians and (in parenthesis) first and third quartiles, according to data normality. ADL: Activities of Daily Living; IADL: Instrumental Activities of Daily Living; MMSE: Mini Mental State Examination. P values of Wilcoxon analyses are displayed.

* represents significant differences between pre and post data.

**Fig 2:**
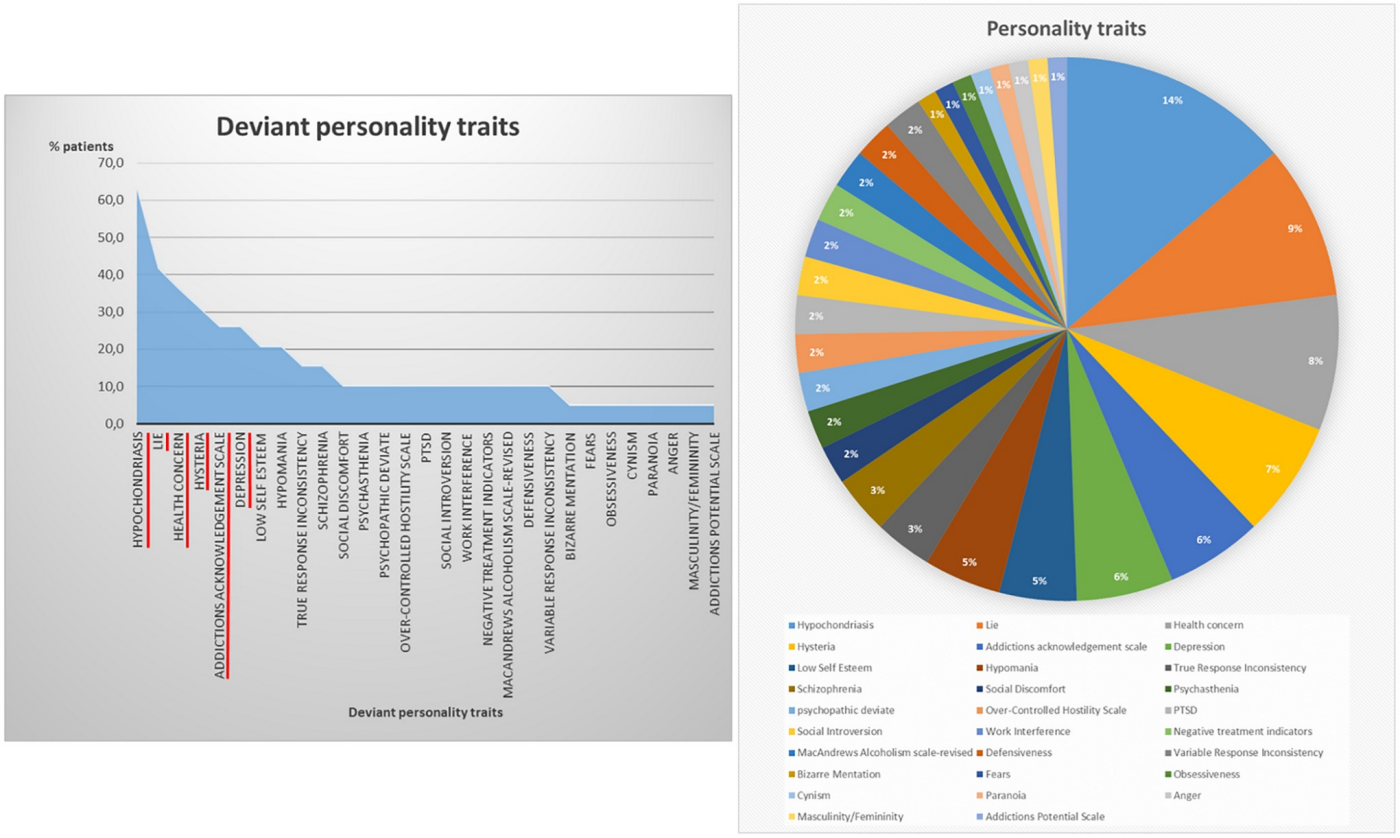
Personality traits. Figure shows the representation of deviant personality traits amongst participants.

The descriptive analysis of the psychological profile of patients (MMPI-2) showed that 63.2% of patients presented with scores above normal for hypochondriasis. Scores above normal were also observed for lie (42.1%), health concern (36.8%), hysteria (31.6%), addictions acknowledgement scale (26.3%) and depression (26.3%). Detailed results of psychological profile are displayed in Fig 2.

### Participation, function and proprioception

Results are exposed in Table 5. The statistical analyses of RMDQ and kinematics data revealed a significant improvement in participation (RMDQ, p<0.001), trunk functionality (Maximal range of motion − Rotation, p = 0.002; Average range of motion − Rotation, p = 0.008), and proprioception (Repetition Index − Rotation, p = 0.024). The scale RMDQ reached also a clinical relevance of change, improving more than its MCID set at 5 points in chronic low-back pain [59].

**Table 5:** Physical therapy evaluations.

Tests		Pre	Post	P value
RMDQ		14.07 ± 4.59	8.28 ± 6.49	< 0.001 *
Kinematic Data	Maximal Rotation	52.92 ± 22.26	69.39 ± 17.43	0.002 *
	Average Rotation	33.55 ± 20.32	53.20 ± 20.94	0.008 *
	Repetition Index	0.59 ± 0.15	0.74 ± 0.17	0.024 *

Table shows pre and post average values (± standard deviations) for participation (RMDQ), function (Maximal and Average Rotation) and proprioception (Repetition Index). RMDQ: Roland and Morris Disability Questionnaire.

* represents significant differences between pre and post data (t-test).

The improvements in participation (RMDQ) correlated with the improvements reported by the patients on the GIC scale (Pearson R = 0.592, p = 0.006), NRS (Pearson R = 0.598, p = 0.006), MPQ PRI-TOT (Pearson R = 0.711, p<0.001) and MPQ NWC (Pearson R = 0.629, p = 0.003).

## Discussion

In this proof-of-concept study, we were able to show that VR-based rehabilitation could have significant impact on chronic low-back pain. Indeed, after six weeks of treatment, significant decreases were observed for all pain scores, some of which overstepping the MCID reported in the literature. This decreased pain sensation was also accompanied by improvements in QoL, in some cognitive functions and sensorimotor output. Although such results need to be reproduced in a larger, controlled, randomized study, we believe these data bring evidence on the fact that virtual reality can be efficient in helping chronic low-back pain patients in regaining better motor function together with a reduced pain sensation and improvement in their quality of life.

Our treatment was based on the hypothesis that helping patients to regain a correct body perception would help them improve their functional abilities, improve their QoL and reduce pain sensations. The fact that patients improved significantly in movement reproducibility and amplitude with training seems to indicate that VR-based rehabilitation produced significant proprioceptive and functional improvements. Previous studies also showed that VR could improve movement and body position perception [28]. In chronic neuropathic pain, a VR-based treatment could induce analgesia in association with an improved embodiment sensation [60].

Although further studies will need to investigate the corticospinal mechanisms induced by VR-based rehabilitation, we could hypothesize that such effects might have been due to corticospinal reorganization. Indeed, VR training has influences on various cortical and subcortical neuronal circuits, particularly related to learning. First, in some simple motor rehabilitation exercises, the motor copy strategies use mirror neurons system to improve learning [26]. Moreover, thanks to the online and final feedbacks given to the subject during VR training, the participant has a developed knowledge of results and the knowledge of her/his performance, which constitutes the basis for reinforcement learning where the cortico-basal ganglia circuits play a central role [27]. Lastly, the presence of virtual teachers and guidance engage another type of learning: the supervised learning where the cerebellum plays a central role [28]. A small open-label study based on the same hypothesis already reported improvement in pain sensation in patients suffering from complex regional pain syndrome [61]. The authors showed that 5 sessions of virtual reality mirror visual feedback therapy was able to produce >50% reduction in pain intensity. Moreover, neuroplastic changes due to VR training has been recently demonstrated in patients presenting with chronic neglect [60]. In these patients, five weeks of VR-based training improved their performance in the Posner’s cuing task. These improvements were accompanied by increased BOLD signal changes in the Dorsolateral Prefrontal Cortex, the Anterior Cingulate Cortex and the bilateral Temporal Cortex, which are areas related to the top-down component of the attention task. Virtual reality has also been used to induce “full body illusions” during which visuo-tactile conflicts can induce one subject to feel the ownership for a virtual body [62,63]. Such approach has been used to reduce chronic pain sensation in a heterogeneous group of chronic pain patients experiencing full body illusions [64]. The authors hypothesized that a visual analgesic effect might have been caused by viewing the body, although other mechanisms might have been involved as well since there was a significant correlation between the pain reduction and the synchrony of stimulations.

Other non-pharmacological therapies have been shown to improve chronic pain. In their review, Chou and Huffman found good evidence that psychological interventions (cognitive-behavioral therapy and progressive relaxation), exercise, interdisciplinary rehabilitation, functional restoration and spinal manipulation were effective for chronic low-back pain. These interventions were associated with moderate effects such as 10 to 20 points difference on a 100-point visual analogue pain scale or 2 to 4 points on the RMDQ [21]. Interestingly, our approach reduced the median NRS score of 4.5 points and reduced the average RMDQ score of 6 points (both results overstepping the MCID). These results underline the potential robust effect of this VR-based treatment for chronic low back pain.

Cognitive-behavioral treatments for chronic pain aim to reduce feelings of helplessness and uncontrollability and instate behaviors that limit the impact of pain on QoL. Cognitive-behavioral approaches also focus on extinguishing pain behaviors and acquiring healthy ones. Such treatments can modulate brain activity related to pain. Indeed, it has been shown that exposure therapy can alter brain processes related to stimuli that are relevant for the disorder. Sensory approaches can also modulate cortical organization. This has been shown in phantom limb pain which was decreased by somatosensory training acuity. Motor strategies, such as mirror therapy and virtual limbs for neuropathic pain might correct cortical body maps to remove the incongruence between motor commands and sensory feedback (for review see [65]).

The chronic pain state can trigger a cascade of changes in psychological processes [66] that seem to qualify, according to our results, for a treatment with VR. Indeed, previous studies demonstrated that chronic pain can affect various cognitive functions, such as attention, verbal memory and executive functions [5]. In line with these reports, our results showed significant improvements in short- and long-term verbal memory, attention, language and long-term visuospatial memory after treatment, with significant correlations between cognitive improvements and reduction of pain sensation. There was no significant improvement in other measurements of attention (matrices, TMT) or short-term visuospatial memory, working memory, problem solving and other executive functions. One can hypothesize that the duration of treatment might have been too short and that longer treatments might have had broader cognitive effects. Further studies should be dedicated to the investigation of the relationships between VR training, pain sensation, cortical reorganization and cognitive functions. Our results demonstrated that this VR-based treatment was able to act on the multidimensional aspects of pain, improving pain sensations, cognitive functions, together with functional and proprioceptive aspects. The combination of all these multidimensional aspects might have been responsible for the improvements in various subdomains of QoL, such as physical functioning and physical role functioning, vitality, social role functioning.

In line with previous reports, our patients presented with abnormal levels of hypochondria, depression, hysteria and health concerns, as shown by the MMPI analyses [6–9]. The MMPI has been used to classify psychological traits of patients with chronic pain. These personality traits of hypochondria, depression and hysteria have been shown to play a role in the chronicization of pain [67] and may increase the affective dimension of pain [68]. Although previous studies reported increased anxiety in chronic pain patients [6,7], our MMPI measurements revealed abnormal anxiety traits in only 11% of our population. More investigations on anxiety and chronic pain are thus needed to better investigate the relationship between both factors. Indeed, further studies should investigate how personality profile might influence non-pharmacological treatments of chronic pain in order to provide patients with better personalized treatments.

Chronic pain has become a major health concern worldwide. Pharmaceutical companies are currently addressing the crisis of prescription drug abuse or misuse through the development of abuse-deterrent formulations of opioid analgesics, addiction treatments, medication to treat opioids overdoses, and non-opioid drugs [69]. The development of alternative, non-pharmacological treatments is essential to offer patients the best personalized care and to reduce the risk of drug misuse. Non-pharmacological treatments could also present the advantage of a good compliance amongst patients. In particular, VR training has been reported as a pleasant treatment [24]. Our results confirm this statement as all of our patients underwent the full training without dropouts.

Our results open thus new study perspective and further randomized studies are necessary to define: (1) treatment effectiveness; (2) the best treatment duration; (3) in which manner might these improvements affect patients’ motor and functional activities; (4) how such VR treatments can reduce analgesic drug intake; (5) what are the benefits of this kind of treatment with respect to the traditional ones. We acknowledge the absence of a control group in this study, the heterogeneity in patients’ profile, as well as an absence of correction for multiple hypothesis testing.

Our study showed that a virtual reality-based motor training was able to reduce chronic pain and act on the multidimensional aspects of pain, such as pain sensation itself, quality of life, sensory and motor sensations as well as cognitive functions. These data serve as a proof-of-concept study that should lead to larger, controlled, randomized clinical trials to better investigate the potential of virtual reality to act on body perception to treat chronic pain.

## References

[1] Treede R-D, Rief W, Barke A, Aziz Q, Bennett MI, Benoliel R, et al A classification of chronic pain for ICD-11. Pain. 2015;156: 1003–1007. 25844555 10.1097/j.pain.0000000000000160 PMC4450869

[2] Baykara RA, Bozgeyik Z, Akgul O, Ozgocmen S. Low back pain in patients with rheumatoid arthritis: clinical characteristics and impact of low back pain on functional ability and health related quality of life. J Back Musculoskelet Rehabil. 2013;26: 367–374. 10.3233/BMR-130393 23948822

[3] Weiss AL, Ehrhardt KP, Tolba R. Atypical Facial Pain: a Comprehensive, Evidence-Based Review. Curr Pain Headache Rep. 2017;21: 8 10.1007/s11916-017-0609-9 28251523

[4] Harstall C, Ospina M. How Prevalent Is Chronic Pain? Pain Clin Updat Int Assoc Study Pain. 2003;XI: 1–4.

[5] Moriarty O, McGuire BE, Finn DP. The effect of pain on cognitive function: a review of clinical and preclinical research. Prog Neurobiol. 2011;93: 385–404. 10.1016/j.pneurobio.2011.01.002 21216272

[6] Mongini F, Ibertis F, Barbalonga E, Raviola F. MMPI-2 profiles in chronic daily headache and their relationship to anxiety levels and accompanying symptoms. Headache. 2000;40: 466–472. 10849043 10.1046/j.1526-4610.2000.00070.x

[7] Slesinger D, Archer RP, Duane W. MMPI-2 characteristics in a chronic pain population. Assessment. 2002;9: 406–414. 10.1177/1073191102238153 12462761

[8] Applegate KL, Keefe FJ, Siegler IC, Bradley LA, McKee DC, Cooper KS, et al Does personality at college entry predict number of reported pain conditions at mid-life? A longitudinal study. J Pain Off J Am Pain Soc. 2005;6: 92–97. 10.1016/j.jpain.2004.11.001 15694875

[9] Kato F, Abe T, Kanbara K, Ban I, Kiba T, Kawashima S, et al Pain threshold reflects psychological traits in patients with chronic pain: a cross-sectional study. Biopsychosoc Med. 2017;11: 13 10.1186/s13030-017-0098-4 28507594 PMC5429533

[10] Leadley RM, Armstrong N, Reid KJ, Allen A, Misso KV, Kleijnen J. Healthy aging in relation to chronic pain and quality of life in Europe. Pain Pract Off J World Inst Pain. 2014;14: 547–558. 10.1111/papr.12125 24138082

[11] Park J, Hughes AK. Nonpharmacological approaches to the management of chronic pain in community-dwelling older adults: a review of empirical evidence. J Am Geriatr Soc. 2012;60: 555–568. 10.1111/j.1532-5415.2011.03846.x 22288789

[12] Andrew R, Derry S, Taylor RS, Straube S, Phillips CJ. The costs and consequences of adequately managed chronic non-cancer pain and chronic neuropathic pain. Pain Pract Off J World Inst Pain. 2014;14: 79–94. 10.1111/papr.12050 23464879

[13] Flor H, Braun C, Elbert T, Birbaumer N. Extensive reorganization of primary somatosensory cortex in chronic back pain patients. Neurosci Lett. 1997;224: 5–8. 9132689 10.1016/s0304-3940(97)13441-3

[14] Lewis JS, Kersten P, McCabe CS, McPherson KM, Blake DR. Body perception disturbance: a contribution to pain in complex regional pain syndrome (CRPS). Pain. 2007;133: 111–119. 17509761 10.1016/j.pain.2007.03.013

[15] Förderreuther S, Sailer U, Straube A. Impaired self-perception of the hand in complex regional pain syndrome (CRPS). Pain. 2004;110: 756–761. 15288417 10.1016/j.pain.2004.05.019

[16] Moseley GL. I can’t find it! Distorted body image and tactile dysfunction in patients with chronic back pain. Pain. 2008;140: 239–243. 18786763 10.1016/j.pain.2008.08.001

[17] Flor H, Elbert T, Knecht S, Wienbruch C, Pantev C, Birbaumer N, et al Phantom-limb pain as a perceptual correlate of cortical reorganization following arm amputation. Nature. 1995;375: 482–484. 10.1038/375482a0 7777055

[18] Yang TT, Gallen C, Schwartz B, Bloom FE, Ramachandran VS, Cobb S. Sensory maps in the human brain. Nature. 1994;368: 592–593. 10.1038/368592b0 8145842

[19] Juottonen K, Gockel M, Silén T, Hurri H, Hari R, Forss N. Altered central sensorimotor processing in patients with complex regional pain syndrome. Pain. 2002;98: 315–323. 12127033 10.1016/S0304-3959(02)00119-7

[20] Maihöfner C, Handwerker HO, Neundörfer B, Birklein F. Cortical reorganization during recovery from complex regional pain syndrome. Neurology. 2004;63: 693–701. 10.1212/01.wnl.0000134661.46658.b0 15326245

[21] Chou R, Huffman LH, American Pain Society, American College of Physicians. Nonpharmacologic therapies for acute and chronic low back pain: a review of the evidence for an American Pain Society/American College of Physicians clinical practice guideline. Ann Intern Med. 2007;147: 492–504. 17909210 10.7326/0003-4819-147-7-200710020-00007

[22] Kizony R, Katz N, (Tamar) Weiss PL. Adapting an immersive virtual reality system for rehabilitation. J Vis Comput Animat. 2003;14: 261–268. 10.1002/vis.323

[23] Jack D, Boian R, Merians AS, Tremaine M, Burdea GC, Adamovich SV, et al Virtual reality-enhanced stroke rehabilitation. IEEE Trans Neural Syst Rehabil Eng Publ IEEE Eng Med Biol Soc. 2001;9: 308–318. 10.1109/7333.948460 11561668

[24] “Skip” Rizzo A, Kim GJ. A SWOT Analysis of the Field of Virtual Reality Rehabilitation and Therapy. Presence Teleoperators Virtual Environ. 2005;14: 119–146. 10.1162/1054746053967094

[25] Thornton M, Marshall S, McComas J, Finestone H, McCormick A, Sveistrup H. Benefits of activity and virtual reality based balance exercise programmes for adults with traumatic brain injury: perceptions of participants and their caregivers. Brain Inj. 2005;19: 989–1000. 10.1080/02699050500109944 16263641

[26] Kommalapati R, Michmizos KP. Virtual reality for pediatric neuro-rehabilitation: adaptive visual feedback of movement to engage the mirror neuron system. Conf Proc Annu Int Conf IEEE Eng Med Biol Soc IEEE Eng Med Biol Soc Annu Conf. 2016;2016: 5849–5852. 10.1109/EMBC.2016.7592058 28269584

[27] Ito M, Doya K. Multiple representations and algorithms for reinforcement learning in the cortico-basal ganglia circuit. Curr Opin Neurobiol. 2011;21: 368–373. 10.1016/j.conb.2011.04.001 21531544

[28] Solouki S, Pooyan M. Arrangement and Applying of Movement Patterns in the Cerebellum Based on Semi-supervised Learning. Cerebellum Lond Engl. 2016;15: 299–305. 10.1007/s12311-015-0695-3 26109488

[29] Harvie DS, Smith RT, Hunter EV, Davis MG, Sterling M, Moseley GL. Using visuo-kinetic virtual reality to induce illusory spinal movement: the MoOVi Illusion. PeerJ. 2017;5: e3023 10.7717/peerj.3023 28243537 PMC5324774

[30] Jones T, Moore T, Choo J. The Impact of Virtual Reality on Chronic Pain. PloS One. 2016;11: e0167523 10.1371/journal.pone.0167523 27997539 PMC5172565

[31] Yilmaz Yelvar GD, Çırak Y, Dalkılınç M, Parlak Demir Y, Guner Z, Boydak A. Is physiotherapy integrated virtual walking effective on pain, function, and kinesiophobia in patients with non-specific low-back pain? Randomised controlled trial. Eur Spine J Off Publ Eur Spine Soc Eur Spinal Deform Soc Eur Sect Cerv Spine Res Soc. 2017;26: 538–545. 10.1007/s00586-016-4892-7 27981455

[32] Katz S, Ford AB, Moskowitz RW, Jackson BA, Jaffe MW. STUDIES OF ILLNESS IN THE AGED. THE INDEX OF ADL: A STANDARDIZED MEASURE OF BIOLOGICAL AND PSYCHOSOCIAL FUNCTION. JAMA. 1963;185: 914–919. 14044222 10.1001/jama.1963.03060120024016

[33] Lawton MP, Brody EM. Assessment of older people: self-maintaining and instrumental activities of daily living. The Gerontologist. 1969;9: 179–186. 5349366

[34] Folstein MF, Folstein SE, McHugh PR. “Mini-mental state”. A practical method for grading the cognitive state of patients for the clinician. J Psychiatr Res. 1975;12: 189–198. 1202204 10.1016/0022-3956(75)90026-6

[35] John Raven J. Raven Progressive Matrices. Handbook of Nonverbal Assessment. Springer, Boston, MA; 2003 pp. 223–237. 10.1007/978-1-4615-0153-4_11

[36] De Renzi E, Vignolo LA. The token test: A sensitive test to detect receptive disturbances in aphasics. Brain J Neurol. 1962;85: 665–678. 10.1093/brain/85.4.665 14026018

[37] Novelli G. P. C., Papagno C, Capitani E, Laiacona M, Vallar G, Cappa S. Tre test clinici di ricerca e produzione lessicale. Taratura su soggetti normal. Arch Psicol Neurol Psichiatr. 1986;47: 477–506.

[38] Miceli G, Laudanna A, Burani C, Capasso R. Batteria per l’analisi dei deficit afasici. B.A.D.A. 1994; https://www.iris.unisa.it/handle/11386/3828878?mode=full.19#.W1CYCvkzaUk

[39] Kaplan E, Goodglass H, Weintraub S, Goodglass H. Boston naming test. Philadelphia: Lea & Febiger; 1983.

[40] Orsini A, Grossi D, Capitani E, Laiacona M, Papagno C, Vallar G. Verbal and spatial immediate memory span: normative data from 1355 adults and 1112 children. Ital J Neurol Sci. 1987;8: 539–548. 3429213 10.1007/BF02333660

[41] Wechsler D. A Standardized Memory Scale for Clinical Use. J Psychol. 1945;19: 87–95. 10.1080/00223980.1945.9917223

[42] Corsi PM. Human Memory and the Medial Temporal Region of the Brain. Diss Abstr Int. 1972;34.

[43] Carlesimo GA, Caltagirone C, Gainotti G. The Mental Deterioration Battery: normative data, diagnostic reliability and qualitative analyses of cognitive impairment. The Group for the Standardization of the Mental Deterioration Battery. Eur Neurol. 1996;36: 378–384. 8954307 10.1159/000117297

[44] Reitan RM. Investigation of the validity of Halstead’s measures of biological intelligence. AMA Arch Neurol Psychiatry. 1955;73: 28–35. 13217520 10.1001/archneurpsyc.1955.02330070030005

[45] Jensen AR, Rohwer WD. The Stroop color-word test: a review. Acta Psychol (Amst). 1966;25: 36–93. 5328883 10.1016/0001-6918(66)90004-7

[46] Heaton R, Chelune G, Talley J, Kay G. Wisconsin Card Sorting Test Manual: Revised and expanded. 1993rd ed. Odessa, FL: Psychological Assessment Resources Inc;

[47] Sellbom M, Ben-Porath YS, Bagby RM. Personality and psychopathology: mapping the MMPI-2 Restructured Clinical (RC) Scales onto the Five Factor Model of personality. J Personal Disord. 2008;22: 291–312. 10.1521/pedi.2008.22.3.291 18540801

[48] Beck A.T., Steer R.A., Brown G. Manual for the Beck Depression Inventory-II. San Antonio, TX: Psychological Corporation; 1996.

[49] Ware JE, Sherbourne CD. The MOS 36-item short-form health survey (SF-36). I. Conceptual framework and item selection. Med Care. 1992;30: 473–483. 1593914

[50] McHorney CA, Ware JE, Lu JF, Sherbourne CD. The MOS 36-item Short-Form Health Survey (SF-36): III. Tests of data quality, scaling assumptions, and reliability across diverse patient groups. Med Care. 1994;32: 40–66. 8277801 10.1097/00005650-199401000-00004

[51] Hartrick CT, Kovan JP, Shapiro S. The numeric rating scale for clinical pain measurement: a ratio measure? Pain Pract Off J World Inst Pain. 2003;3: 310–316. 10.1111/j.1530-7085.2003.03034.x 17166126

[52] Melzack R. The McGill Pain Questionnaire: major properties and scoring methods. Pain. 1975;1: 277–299. 1235985 10.1016/0304-3959(75)90044-5

[53] Cleeland CS, Ryan KM. Pain assessment: global use of the Brief Pain Inventory. Ann Acad Med Singapore. 1994;23: 129–138. 8080219

[54] Roland M, Morris R. A study of the natural history of back pain. Part I: development of a reliable and sensitive measure of disability in low-back pain. Spine. 1983;8: 141–144. 6222486 10.1097/00007632-198303000-00004

[55] Roosink M, McFadyen BJ, Hébert LJ, Jackson PL, Bouyer LJ, Mercier C. Assessing the perception of trunk movements in military personnel with chronic non-specific low back pain using a virtual mirror. PloS One. 2015;10: e0120251 10.1371/journal.pone.0120251 25799009 PMC4370585

[56] Willigenburg NW, Kingma I, van Dieën JH. Center of pressure trajectories, trunk kinematics and trunk muscle activation during unstable sitting in low back pain patients. Gait Posture. 2013;38: 625–630. 10.1016/j.gaitpost.2013.02.010 23473809

[57] Farrar JT, Young JP, LaMoreaux L, Werth JL, Poole RM. Clinical importance of changes in chronic pain intensity measured on an 11-point numerical pain rating scale. Pain. 2001;94: 149–158. 11690728 10.1016/S0304-3959(01)00349-9

[58] Macedo LG, Latimer J, Maher CG, Hodges PW, McAuley JH, Nicholas MK, et al Effect of Motor Control Exercises Versus Graded Activity in Patients With Chronic Nonspecific Low Back Pain: A Randomized Controlled Trial. Phys Ther. 2012;92: 363–377. 10.2522/ptj.20110290 22135712

[59] Maughan EF, Lewis JS. Outcome measures in chronic low back pain. Eur Spine J Off Publ Eur Spine Soc Eur Spinal Deform Soc Eur Sect Cerv Spine Res Soc. 2010;19: 1484–1494. 10.1007/s00586-010-1353-6 20397032 PMC2989277

[60] Ekman U, Fordell H, Eriksson J, Lenfeldt N, Wåhlin A, Eklund A, et al Increase of frontal neuronal activity in chronic neglect after training in virtual reality. Acta Neurol Scand. 2018;138: 284–292. 10.1111/ane.12955 29770439

[61] Sato K, Fukumori S, Matsusaki T, Maruo T, Ishikawa S, Nishie H, et al Nonimmersive virtual reality mirror visual feedback therapy and its application for the treatment of complex regional pain syndrome: an open-label pilot study. Pain Med Malden Mass. 2010;11: 622–629. 10.1111/j.1526-4637.2010.00819.x 20202141

[62] Ehrsson HH. The experimental induction of out-of-body experiences. Science. 2007;317: 1048 10.1126/science.1142175 17717177

[63] Lenggenhager B, Tadi T, Metzinger T, Blanke O. Video ergo sum: manipulating bodily self-consciousness. Science. 2007;317: 1096–1099. 10.1126/science.1143439 17717189

[64] Pamment J, Aspell JE. Putting pain out of mind with an ‘out of body’ illusion. Eur J Pain. 2017;21: 334–342. 10.1002/ejp.927 27509229

[65] Moseley GL, Gallace A, Spence C. Bodily illusions in health and disease: physiological and clinical perspectives and the concept of a cortical “body matrix”. Neurosci Biobehav Rev. 2012;36: 34–46. 10.1016/j.neubiorev.2011.03.013 21477616

[66] Simons LE, Elman I, Borsook D. Psychological processing in chronic pain: a neural systems approach. Neurosci Biobehav Rev. 2014;39: 61–78. 10.1016/j.neubiorev.2013.12.006 24374383 PMC3944001

[67] Mongini F, Rota E, Deregibus A, Mura F, Francia Germani A, Mongini T. A comparative analysis of personality profile and muscle tenderness between chronic migraine and chronic tension-type headache. Neurol Sci Off J Ital Neurol Soc Ital Soc Clin Neurophysiol. 2005;26: 203–207. 10.1007/s10072-005-0462-1 16193246

[68] Mongini F, Rota E, Evangelista A, Ciccone G, Milani C, Ugolini A, et al Personality profiles and subjective perception of pain in head pain patients. Pain. 2009;144: 125–129. 19394764 10.1016/j.pain.2009.03.026

[69] Cohen JP, Mendoza M, Roland C. Challenges Involved in the Development and Delivery of Abuse-deterrent Formulations of Opioid Analgesics. Clin Ther. 2018;40: 334–344. 10.1016/j.clinthera.2018.01.003 29398162

